# Piloting an evidence-based intervention for HIV prevention among street youth in Eldoret, Kenya

**DOI:** 10.1007/s00038-020-01349-8

**Published:** 2020-04-09

**Authors:** L. Embleton, E. Di Ruggiero, C. H. Logie, D. Ayuku, P. Braitstein

**Affiliations:** 1 Institute of Medical Science, Faculty of Medicine, University of Toronto, Toronto, Canada; 2 Dalla Lana School of Public Health, University of Toronto, Toronto, Canada; 3 Factor-Inwentash Faculty of Social Work, University of Toronto, Toronto, Canada; 4 Department of Behavioural Science, College of Health Science, Moi University, Eldoret, Kenya; 5 Academic Model Providing Access to Healthcare, Eldoret, Kenya; 6 School of Medicine, College of Health Sciences, Moi University, Eldoret, Kenya

**Keywords:** HIV, Condom use, Evidence-based intervention, Street youth, Kenya, Eldoret, Sexual practices

## Abstract

**Objectives:**

This study presents findings from piloting an adapted evidence-based intervention, Stepping Stones and Creating Futures, to change street-connected young people’s HIV knowledge, condom-use self-efficacy, and sexual practices.

**Methods:**

Eighty street-connected young people participated in a pre- and post-test mixed methods design in Eldoret, Kenya. The primary outcome of interest was HIV knowledge. Secondary outcomes included condom-use self-efficacy and sexual practices. Multiple linear regression models for change scores with adjustment for socio-demographic variables were fitted. Qualitative and quantitative findings are presented together, where integration confirms, expands on, or uncovers discordant findings.

**Results:**

Participants had a significant increase in HIV knowledge from pre- to post-intervention. The median HIV knowledge score pre-intervention was 11 (IQR 8–13) and post-intervention 14 (IQR 12–16). Attendance was significantly associated with HIV knowledge change scores. Qualitatively participants reported increased HIV and condom-use knowledge and improved condom-use self-efficacy and health-seeking practices.

**Conclusions:**

Our findings support the potential for further testing with a rigorous study design to investigate how best to tailor the intervention, particularly by gender, and increase the overall effectiveness of the program.

**Electronic supplementary material:**

The online version of this article (10.1007/s00038-020-01349-8) contains supplementary material, which is available to authorized users.

## Introduction

In Kenya, street-connected young people (SCY), for whom the streets play a central role in their everyday lives and social identities (Office of the United Nations High Commissioner for Human Rights 2017), engage in sexual practices that increase their exposure to HIV (Kaime-Atterhög et al. 2007; Embleton et al. 2015, 2016; Wachira et al. 2015, 2016). In Eldoret, Kenya, SCY have a STI and HIV prevalence that exceeds that of other young people in Kenya (Winston et al. 2015; Shah et al. 2018; Braitstein et al. 2019). Street-connected young women aged 15–24 have an HIV prevalence almost four times higher than young women nationally (11% vs. 3.0%) (NASCOP 2014; Braitstein et al. 2019) and frequently die due to AIDS (Embleton et al. 2018). Street-connected young women experience sexual and gender-based violence and are highly reliant on young men for security, economic, and material provisioning, and they are often perceived as ‘unable to work’ and/or rely on survival sex (Sorber et al. 2014; Embleton et al. 2015, 2016; Wachira et al. 2015; Winston et al. 2015). SCY are socially and economically marginalized, earning less than 100 Kenyan Shillings (Ksh) (~ 1.00 USD) per day, with young men earning significantly more than young women (Sorber et al. 2014). Furthermore, SCY have low levels of condom use and several misconceptions in relation to HIV and STIs (Embleton et al. 2015, 2016; Winston et al. 2015). Yet, knowledge of HIV is critical in recognizing ones’ vulnerability to acquiring HIV and can increase the likelihood of engaging in preventive practices.

An individual’s perceived susceptibility of acquiring HIV is a necessary, but not sufficient, condition for behaviour change (Rosenstock 1974). Knowledge is also essential for an individual’s self-efficacy to perform an outcome, such as condom use (Closson et al. 2018). Studies have demonstrated that higher levels of accurate HIV knowledge significantly increase an individual’s perceived HIV risk (Bernardi 2002), and greater perception of risk has been linked to higher levels of condom-use self-efficacy (CUSE) (Tenkorang and Maticka-Tyndale 2014). However, condom-use and sexual decision-making is typically under the control of men, due to social norms and gender inequities, which reduce young women’s sexual relationship power and subsequent ability to practice safer sex with male partners (Tenkorang and Maticka-Tyndale 2014; Appiah et al. 2017; Closson et al. 2018). Nonetheless, increased knowledge, awareness of risk, and sexual self-efficacy are important foundational components of behaviour change.

HIV prevention strategies have focused on increasing knowledge and changing attitudes, and sexual practices of adolescents through individual-level behavioural interventions. Behavioural change interventions alone are often insufficient to produce long-term positive effects on outcomes given the structural factors that impact an individual’s ability to enact change (Gupta et al. 2008; Gibbs et al. 2012). Structural factors refer to the social, cultural, economic, legal, and political factors in a context that impact an individual’s ability to engage in HIV prevention (Sumartojo et al. 2000). Outcomes are considerably improved when behavioural and structural interventions are combined (Gupta et al. 2008; Gibbs et al. 2012; Wamoyi et al. 2014).Therefore, combined interventions may be a viable approach to changing SCY’s sexual practices and vulnerability to acquiring HIV (Gupta et al. 2008; Wamoyi et al. 2014).

SCY in Kenya require interventions that provide sexual education and promote safe sexual practices, while addressing structural factors, such as gender inequities and livelihoods. In response, we adapted the Stepping Stones and Creating Futures (Jewkes et al. 2014) programs with SCY in Eldoret, Kenya (Embleton et al. 2019). The present analysis seeks to explain and explore how participation in the adapted intervention changed SCY’s HIV knowledge, CUSE, and sexual practices.

## Methods

### Study design

**Fig. 1:**
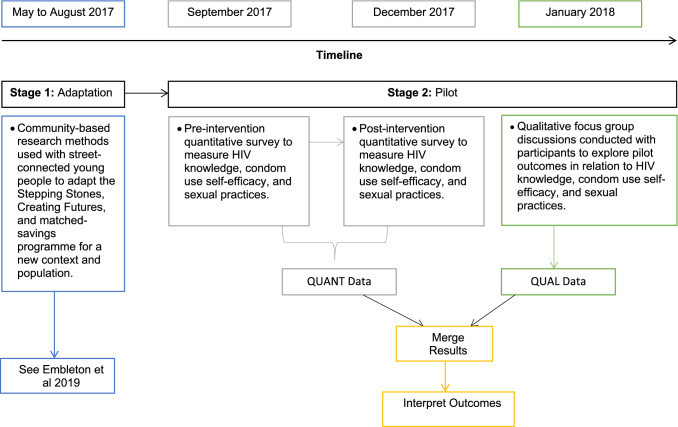
Two stages: study components and methods used in the mixed methods design in Eldoret, Kenya, from 2017 to 2018

From May 2017 to January 2018, a multi-stage mixed methods study was used to adapt and pilot an evidence-based intervention with SCY in Eldoret, Kenya. In the first stage, Stepping Stones and Creating Futures interventions were adapted (Embleton et al. 2019). From September 2017 to January 2018, we piloted the adapted intervention with 80 SCY using a pre- and post-intervention mixed methods design (Fetters et al. 2013). The convergent mixed methods design (Fig. 1) was used to understand and explore the pilot’s outcomes (Fetters et al. 2013; Zhang and Watanabe-Galloway 2014).

### Study setting

This study occurred in Eldoret, Kenya, at the MTRH-Rafiki Centre for Excellence in Adolescent Health. Eldoret is home to Moi University, Moi Teaching and Referral Hospital (MTRH), and the Academic Model Providing Access to Healthcare (AMPATH) (Einterz et al. 2007), a long-standing partnership between Moi University, MTRH, and a consortium of universities. In 2016, there were 1419 SCY under the age of 29 in Eldoret. Of those, 46% (*n *= 653) were aged 15 to 24 years (Braitstein et al. 2019).

### Intervention description

Stepping Stones is a behavioural intervention focused on gender, HIV, and relationship skills (Welbourn 1995; Gordon et al. 2017). Creating Futures is a structural intervention designed to build on Stepping Stones (Jewkes et al. 2014). In South Africa, the combined program was delivered over 12 weeks with out-of-school youth in informal settlements (Jewkes et al. 2014). Our adapted program included 24 sequential sessions over the course of 14 weeks (Embleton et al. 2019); 8 weeks of Stepping Stones followed by 6 weeks of *Kujijenga Kimaisha* (Creating Futures). The Stepping Stones program covered topics on: communication, gender norms, love, sexual and reproductive health, HIV and STIs, condom use, gender-based violence, drug and alcohol use, and peer pressure. The *Kujijenga Kimaisha* program had participants reflect on their livelihood skills and resources and examine how they can strengthen them. The program aimed to support and encourage participants to set an achievable personal goal, and to work towards commencing income-generating activity or training course of their choice.

Each intervention session lasted from 1.5 to 3 h and was conducted in a private tent outside of the adolescent-friendly clinic. Peer facilitators of the same gender facilitated the program under the supervision of the principal investigator (PI) (LE), in single-gender age-stratified groups of 20 participants (ages 16–19 and 20–24). The peer facilitators were respected and trusted members of the street community (Embleton et al. 2019), who underwent 1 month of training on the intervention, facilitation, research conduct, and data collection for this study.

### Study participants

SCY were eligible to participate in the pilot intervention if they were: (1) aged 16–24 years, (2) had spent a portion or majority of their time on the streets for the past 6 months, and (3) were not enrolled in or attending school.

### Sample size

A sample size of 80 participants was determined based on calculations to detect significant mean difference in our primary outcome of HIV knowledge (*α *= 0.05, *β *= 0.20, power = 0.8, SD = 20) by estimating high, medium, and low baseline scores and hypothesized percentage increase in score (25%, 15%, or 10%). As this was a pilot and feasibility study (Thabane et al. 2010), it was not powered to detect changes in secondary outcomes.

### Participant recruitment and enrolment

Due to an existing relationship between the research team and SCY, the locations where SCY live and congregate in Eldoret were known, and outreach was conducted in these locations to explain the purpose of the research and the intervention. Peer facilitators created four age- and sex-stratified sampling lists of SCY who met the eligibility criteria and who indicated their interest in participating in the intervention. Participants were selected using a simple random sampling procedure generating random numbers. Selected participants were invited to enrol at the adolescent-friendly clinic. If a selected participant declined to participate or did not come on the enrolment date, another participant from the list was randomly selected until the desired sample size of 80 participants was reached, consisting of 20 participants for each age- and gender-stratified group (ages 16–19 and 20–24).

### Study procedures

Consenting participants completed a paper-based standardized questionnaire administered by the peer facilitators. Pre-intervention surveys were administered face to face in Swahili in a private tent under the supervision of the PI. Throughout the intervention’s implementation, attendance records were kept to monitor retention and participation in the intervention. In week 14, participants completed a post-intervention survey administered by the peer facilitators. Of the 80 participants that completed pre-intervention surveys, 67 completed post-intervention surveys. At post-intervention, six participants from each of the intervention groups were purposively invited to participate in focus group discussions (FGDs) about their experience in the program. Twenty-one participants returned to attend FGDs, which were conducted in Swahili by a trained peer facilitator and the PI. FGDs took 1 to 1.5 h and occurred in a private tent.

### Measures

Pre- and post-intervention standardized questionnaires were pretested for use with five male and five female SCY aged 16–24 who provided feedback regarding the clarity and acceptability of the questions. Minor changes to the Swahili wording of questions were made. The questionnaire captured basic socio-demographic data including: age, school attendance, relationship status, orphan status, time typically spent on the streets, time street-involved, and economic resources. The primary outcome of interest, HIV knowledge, was selected due to substantial misconceptions about HIV in the street subculture (Embleton et al. 2016). As well, HIV knowledge is an evidence-based outcome of the Stepping Stones intervention (Skevington et al. 2013). HIV knowledge was assessed using the 18-item (Cronbach’s *α *= 0.60) HIV-KQ-18 questionnaire (Carey and Schroeder 2002). HIV-KQ-18 answer options are ‘true’, ‘false’, or ‘don’t know’, and each correct answer is given one point; scores are then summed. Higher scores represent greater HIV knowledge (Carey and Schroeder 2002). Secondary outcomes included CUSE and sexual practices. CUSE was measured using the 9-item validated (Cronbach’s *α *= 0.73) condom-use self-efficacy scale—Ethiopia (Shaweno and Tekletsadik 2013). Responses for each item were scored as 0 = strongly disagree, 1 = disagree, 2 = undecided, 3 = agree, and 4 = strongly agree. Items that were negatively worded were reverse coded. Higher scores indicate greater CUSE (Shaweno and Tekletsadik 2013). Sexual practices were measured by asking participants the following questions: ever engaged in vaginal sex, age of first vaginal sex, was the first vaginal intercourse voluntary, last vaginal sex, last vaginal sex condom use, frequency of condom use, number of vaginal sex partners in the past month, ever exchange sex for money, shelter, food, security or other material items, in the past month exchange sex, frequency of exchanging sex, ever tested for HIV, and when was the last HIV test.

FGDs used an interview guide that asked participants about their experience participating in the program, what they learnt in relation to HIV, condom use, and sexual and reproductive health and how the program changed or did not change their sexual practices.

### Analyses

Quantitative and qualitative data were collected and analysed separately. Findings were merged for comparison after statistical and textual analyses of the data (Fetters et al. 2013). Data were then integrated through a weaving approach whereby qualitative and quantitative findings are presented together on a theme-by-theme basis, where integration confirms, expands on, or uncovers discordant findings (Fetters et al. 2013).

Quantitative data collected on paper surveys were checked for errors and missing data and manually entered into Epi Info (version 7.2.1). Data were exported into R Studio for analysis. Categorical variables were summarized using frequencies and percentages. Continuous variables were summarized using either median and interquartile range or mean and standard deviation. A Shapiro–Wilk test and visual analysis of histogram and Q–Q plots were used to test for normality of the outcome variables for HIV knowledge and CUSE scores. Due to the non-normality of the HIV knowledge scores, a Wilcoxon signed-rank test was used to test whether participants had improved their HIV knowledge from pre- to post-intervention. CUSE scores were normally distributed, and a paired *t* test was employed to compare mean pre- and post-test scores. Difference scores were calculated by taking the post-intervention scores minus the pre-intervention scores. Multiple linear regression models were fit to predict HIV knowledge and condom-use self-efficacy change scores. Models were adjusted for the covariates of age, gender, education level, attendance, time street-connected, relationship status, and ever tested for HIV. Pre-intervention scores were controlled for in the models, which ensured the net relationships of the difference score to the independent variables in the model are adjusted for the confounding effects of the pre-intervention score. When pre-intervention scores are incorporated into the equation, concerns and problems regarding difference scores are countered (Dalecki and Willits 1991). Changes in sexual practices were compared using McNemar’s test for paired nominal data.

All participants post-intervention were asked to complete questionnaires. Outcome analyses included all enrolled participants, irrespective of program attendance or completion. Thirteen participants were not interviewed post-intervention: eight were lost to follow-up, one dropped out of the program, and four had moved and could not be located. Baseline scores were carried forward post-intervention for these participants. This provides a conservative estimate of the intervention effect when outcomes are not expected to decline (European Medicines Agency 2011). In total, four single items for four participants were missing from the HIV and CUSE scales. For these, scale items were imputed using the mode (HIV scale) and individual mean (CUSE) (Shrive et al. 2006). To test the accuracy of results using this missing data strategy, a complete case analysis was conducted. There were no differences in outcomes or statistical significance compared to the imputed analyses. We present both complete cases and last value carried forward outcomes in our findings.

Qualitative audio-recorded data were transcribed into Swahili and translated into English. Transcribed and translated data were imported into NVivo software for analysis. A codebook was developed, and qualitative data were analysed using thematic analysis driven by analytic interest (Braun and Clarke 2006) by the PI to explore concepts in relation to how participation in the intervention changed or did not change SCY’s HIV knowledge, CUSE, sexual practices, and other health experiences.

## Results

### Socio-demographics and attendance

The median age of participants was 19.5 years (IQR 17–22) (Table 1). The majority of participants had attended some school (96%). Over half of young women reported being in a relationship (60%), defined as being married or having a girlfriend or boyfriend. In contrast, the majority of young men reported being single (73%). Almost all (85%) participants had been street-connected for greater than 1 year. Less than half (35%) of participants reported that both of their parents were alive. Participants attended a median of 11 sessions (IQR 1–20) over 14 weeks. Attendance differed by gender and within age groups (Supplementary Table 1).

**Table 1:** Socio-demographic characteristics of all participants, those lost to follow-up, and those retained in Eldoret, Kenya, from 2017 to 2018

Socio-demographics	All participants			Lost to follow-up participants	Retained participants
	Total *N *= 80 *n* (%)	Young women *N* = 40 *n* (%)	Young men *N *= 40 *n* (%)	*N *= 13 *n* (%)	*N *= 67 *n* (%)
Age (median, interquartile range)	19.5 (17–22)	19.5 (17–23)	19.5 (17–21)	20 (17–20)	19 (17–22)
Ever attended school					
Yes	77 (96.3)	38 (95.0)	39 (97.5)	12 (92.3)	65 (97.0)
No	3 (3.7)	2 (5.0)	1 (2.5)	1 (7.7)	2 (3.0)
Level of education					
Primary school	66 (82.5)	32 (80.0)	34 (85.0)	10 (77.0)	56 (83.6)
Secondary school	11 (13.7)	6 (15.0)	5 (12.5)	2 (15.0)	9 (13.4)
None	3 (3.8)	2 (5.0)	1 (2.5)	1 (7.7)	2 (3.0)
Highest grade completed					
Primary level (median, interquartile range)	7 (5–7)	6 (5–7)	7 (6–7)	7 (6–8)	7 (5–7)
Secondary level (median, interquartile range)	2 (1–4)	3 (2–3)	2 (2–2)	2 (2–2)	3 (2–3)
Relationship status					
Single	42 (52.4)	13 (32.5)	29 (72.5)	6 (46.2)	36 (53.7)
Have a girlfriend or boyfriend	23 (28.7)	12 (30.0)	11 (27.5)	6 (46.2)	17 (25.4)
Married	12 (15.0)	12 (30.0)	0 (0)	1 (7.7)	11 (16.4)
Divorced	2 (2.5)	2 (5.0)	0 (0)	0 (0)	2 (3.0)
Widowed	1 (1.2)	1 (2.5)	0 (0)	0 (0)	1 (1.5)
Time street-connected					
6 months–1 year	12 (15.0)	8 (20.0)	4 (10.0)	2 (14.9)	10 (15.4)
1–2 years	15 (18.8)	9 (22.5)	6 (15.0)	1 (20.9)	14 (7.7)
2–5 years	32 (40.0)	10 (25.0)	22 (55.0)	8 (35.8)	24 (61.5)
> 5 years	21 (26.2)	13 (32.5)	8 (20.0)	2 (28.4)	19 (28.4)
Time on streets					
Day and night	25 (31.25)	14 (35.0)	11 (27.5)	5 (38.5)	20 (29.9)
Day only	47 (58.7)	24 (60.0)	23 (67.5)	4 (30.8)	43 (64.2)
Varies	7 (8.75)	2 (5.0)	5 (12.5)	4 (30.8)	3 (4.5)
Missing	1 (1.25)	–	1 (2.5)	–	1 (1.5)
Parents vital status					
Mother deceased/vital status unknown	8 (10.0)	5 (12.5)	3 (7.5)	2 (15.4)	6 (9.0)
Father deceased/vital status unknown	29 (36.25)	14 (35.0)	15 (37.5)	3 (23.1)	26 (38.8)
Both parents deceased/vital status unknown	15 (18.75)	7 (17.5)	8 (20.0)	3 (23.1)	12 (17.9)
Both parents alive	28 (35.0)	14 (35.0)	14 (35.0)	5 (38.5)	23 (34.3)
Amount earned/day					
< 100 Kenyan Shillings	27 (33.8)	22 (55)	5 (12.5)	5 (38.5)	22 (32.8)
> 100 Kenyan Shillings	53 (66.3)	18 (45)	35 (87.5)	8 (61.5)	45 (67.2)

### HIV knowledge

Participants had a significant increase in HIV knowledge from pre- to post-intervention (Table 2). The median HIV knowledge score pre-intervention was 11 (IQR 8–13) and post-intervention 14 (IQR 12–16) (*p* < 0.0001). One young woman aged 16–19 stated that prior to the intervention she ‘*didn’t know about HIV*’, while a young man aged 20–24 changed his perceptions about what HIV is: ‘*I realized that what I initially thought about HIV was wrong*’. This expanded to learning about STIs as discussed by a young man:

**Table 2:** Median human immunodeficiency virus knowledge and mean condom-use self-efficacy scores pre- and post-intervention for complete cases and all participants stratified by gender in Eldoret, Kenya, from 2017 to 2018

Score	Pre-intervention				Post-intervention				*p* value^b^
	*N *= 80	Young women *n *= 40	Young men *n *= *n*=40	Baseline retained^a^ *N *= 67	Complete cases *N *= 67	*N *= 80	Young women *N *= 40	Young men *N *= 40	
HIV-KQ-18 median (IQR)	11 (8–13)	11 (9–12)	11 (8–13)	11 (8–13)	15 (13–17)	14 (12–16)	14 (12–15)	16 (12–17)	< 0.001

^a^Participants with both baseline and endline data representing complete cases

^b^Comparing pre-intervention score (*N *= 80) and post-intervention score (*N *= 80)

We didn’t know how diseases like STIs are spread and how we can prevent them, but we learnt how to prevent and treat those diseases. So, health wise it helped us because we learnt how to fight those diseases.

(Young man, 20–24)

The association between participant attendance and changes in HIV knowledge change scores was explored in a multiple linear regression model (Table 3). Participants with a medium level of attendance had a 2.8 point increase (95% CI 1.17–4.37) in mean HIV knowledge change score relative to those with low attendance, while those with high attendance had a 4.2 point increase (95% CI 2.80–5.50) in mean HIV knowledge change score relative to those with low attendance while adjusting for age, gender, education, time street-connected, relationship status, ever being tested for HIV, and HIV knowledge score at baseline.

**Table 3:** Multiple regression models to predict change in human immunodeficiency virus knowledge score in Eldoret, Kenya, from 2017 to 2018

Predictors	HIV knowledge change score *β* and 95% CI
Intercept	6.1 (0.53 to 11.6)*
Age (years)	−0.03 (−0.29 to 0.24)
Gender	
Young woman	Ref
Young man	1.5 (0.18 to 2.74)*
Education level	
Primary/none	Ref
Secondary	1.0 (−0.71 to 2.71)
Attendance (categorical)	
Low	Ref
Medium	2.8 (1.17 to 4.37)***
High	4.2 (2.80 to 5.50)***
Time street-connected	
6 months–1 year	−0.5 (−2.53 to 1.53)
1–2 years	Ref
2–5 years	1.1 (−0.63 to 2.78)
> 5 years	0.9 (−0.96 to 2.74)
Relationship status	
Single/divorced/widowed	−0.5 (−1.80 to 0.85)
Married/girlfriend/boyfriend	Ref
Ever tested for HIV	
Yes	Ref
No	1.3 (−1.23 to 3.82)
CUSE score at baseline	
HIV score at baseline	−0.6 (−0.76 to −0.381)***
*R*^2^	0.53
Adjusted *R*^2^	0.45
*p* value for model	< 0.001

Significant codes: 0 ‘***’ 0.001 ‘**’ 0.01 ‘*’ 0.05 ‘+’ 0.1

There were gender differences in HIV knowledge changes. Young women and young men had a baseline median score of 11. For young women, their median score increased to 14 (IQR 12–15) and young men to 16 (IQR 12–17). Gender was significantly associated with HIV knowledge change score (Table 3). The HIV knowledge change score was 1.5 points (95% CI 0.18–2.74) higher for young men than for the reference group, young women, controlling for other variables.

Qualitative interviews with participants confirmed changes in HIV transmission knowledge. One young woman aged 20–24 stated: ‘*I learnt that you can live with someone who has HIV. You can’t get it from using cups and sharing a bed*’. While a young man aged 16–19 discussed learning about the ability to prevent transmission to children among couples living with HIV: ‘*Because even if either of you have HIV, you can have kids that don’t have it*’.

Young men discussed gaining knowledge regarding the use of post (PEP)- and pre-exposure prophylaxis (PrEP) for HIV prevention:

Let me add something. The experience I got from this program is about PEP and PrEP. Before and after. Like if you go find a girl and have sex with her without protection, you go see the doctor within 72 h. You rush there. You go very fast and get medicine to protect yourself.

(Young man, 20–24)

This knowledge extended to young women understanding about the availability and use of PrEP for discordant couples:

When I came here, I learnt that if your husband has the disease [HIV], you can come and be given medicine [PrEP] to prevent that disease.

(Young woman, 16–19)

Across age and gender strata, participants described that they learnt that condom use could prevent HIV and STI transmission:

I learnt how to use a condom. How you can open the condom and know everyone is not the same. Others may have diseases like STIs or HIV, so you have to protect yourself with a condom. That is what I learnt here. Honestly, I didn’t know, but now I know.

(Young man, 16–19)

### Condom-use self-efficacy, condom use, and sexual practices

Learning about correctly using male and female condoms was a program component, which aimed to improve CUSE and condom use among participants. At baseline, the mean CUSE score was 20.7 (SD 4.6) (Table 4). Post-intervention the mean CUSE score demonstrated a small non-significant increase (21.4, SD 4.3) with similar scores across genders. A multiple linear regression model was fit to predict CUSE score change (Table 5). Despite non-statistically significant changes, qualitative accounts among participants supported an increase in CUSE expanding on intervention outcomes. As one young man reported, the intervention increased his ability to use condoms:

**Table 4:** Mean condom-use self-efficacy scores pre- and post-intervention for complete cases and all participants stratified by gender in Eldoret, Kenya, from 2017 to 2018

Score	Pre-intervention				Post-intervention				*p* value^b^
	*N *= 80	Young women *n *= 40	Young men *n *= 40	Baseline retained^a^ *N *= 67	Complete cases *N *= 67	*N *= 80	Young women *N *= 40	Young men *N *= 40	
CUSE mean (SD)	20.7 (4.6)	20.7 (5.1)	20.7 (4.0)	20.8 (4.9)	21.7 (4.5)	21.4 (4.3)	21.3 (4.9)	21.5 (3.7)	0.2476

^a^Participants with both baseline and endline data representing complete cases

^b^Comparing pre-intervention score (*N *= 80) and post-intervention score (*N *= 80)

**Table 5:** Multiple regression model to predict change in condom-use self-efficacy in Eldoret, Kenya, from 2017 to 2018

Predictors	CUSE change score *β* and 95% CI
Intercept	16.2** (6.5 to 26.0)
Age (years)	0.2 (−0.3 to 0.6)
Gender	
Young woman	Ref
Young man	−0.7 (−2.8 to 1.45)
Education level	
Primary/none	Ref
Secondary	1.4 (−1.7 to 4.5)
Attendance (categorical)	
Low	Ref
Medium	−0.4 (−3.0 to 2.3)
High	0.7 (−1.6 to 2.9)
Time street-connected	
6 months–1 year	−2.2 (−5.7 to 1.3)
1–2 years	Ref
2–5 years	0.7 (−2.2 to 3.6)
> 5 years	−2.7 (−5.9 to 0.4)
Relationship status	
Single/divorced/widowed	−0.05 (−2.2 to 2.1)
Married/girlfriend/boyfriend	Ref
Ever tested for HIV	
Yes	Ref
No	0.9 (−3.4 to 5.2)
CUSE score at baseline	−0.9 (−1.1 to −0.6)***
HIV Score at baseline	
*R*^2^	0.51
Adjusted *R*^2^	0.43
*p* value for model	< 0.001

Significant codes: 0 ‘***’ 0.001 ‘**’ 0.01 ‘*’ 0.05 ‘+’ 0.1

To me this program has helped me and taught me a lot. In the past I wouldn’t have used a condom. It was a burden. But it has helped me. It has taught me how to use a condom. Now I know there are diseases, something I ignored in the past…I can protect myself.

(Young man, 20–24)

Young women also indicated they learnt about female condoms and how to use them:

When we were in the streets, we didn’t even know there were female condoms. We knew there was a female condom but didn’t know how to use it. When we came here is when we were taught and if you find a fellow female in trouble you explain to her how she can protect herself.

(Young woman, 16–19)

The correct use of condoms was another area in which participants’ acquired knowledge through program participation as discussed by a young man:

We learnt that you shouldn’t use two condoms. If you use two it may burst and if the person you were having sex with had HIV, you will be infected. So, we learnt not to use two and how to put one on.

(Young man, 16–19)

In spite of participants’ qualitative accounts of increased knowledge of condoms and CUSE, condom use and other sexual practices remained generally unchanged post-intervention (Supplementary Table 2). Reported condom use at last vaginal sex was low among all participants pre- and post-intervention (35%). Post-intervention there was an increase in the proportion of young men reporting condom use at last sex (28% to 42%), although not statistically significant. Similarly, there was a small non-significant increase in the per cent of male participants reporting using condoms always or most of the time for vaginal sex (14% to 21%), whereas this did not change significantly for young women (23% to 24%).

### Health-seeking practices

Almost all participants reported having been tested for HIV in the past (94%) (Supplementary Table 2). There was a small increase in the proportion of participants who reported being tested in the last 6 months from 71% pre- to 84% post-intervention. One young man discussed how the program helped him to know his HIV status:

This program has helped me in many ways. First knowing my [HIV] status and about my health in general.

(Young man, 20–24)

Participants discussed learning to support their peers and to promote health-seeking practices. This extended to young women giving advice and accompanying their friends to get family planning as one young woman aged 16–19 explained: ‘*I can tell her to let me take her to the hospital to get family planning*’. Finally, both young men and women participants discussed increasing their knowledge in relation to family planning methods:

I learnt what I didn’t know. I learnt about family planning. I heard there is something a girl can put here. I don’t know if it is an injection… Yes. Implant. It protects you for two or three years. I didn’t know that until I came to this program.

(Young man, 20–24)

Overall, these qualitative accounts expanded on the intervention’s impact on participants’ health-seeking practices in relation to HIV testing, peer support, and family planning.

## Discussion

This is the first known study of an evidence-based combination HIV-related intervention for SCY in sub-Saharan Africa. Following the intervention, participants reported an increase in HIV knowledge, our primary outcome of interest. While participants did not have significant changes in secondary outcomes, qualitative accounts suggested an improvement in condom knowledge and CUSE as well as in important sexual and reproductive health-seeking practices. Our pilot findings support the need for additional tailoring of the intervention for gender differences and the potential for additional testing with a more rigorous study design.

We previously documented a myriad of misconceptions regarding the transmission of HIV, STIs, and condom use among SCY in Eldoret, Kenya (Embleton et al. 2015, 2016). Given these previous findings and low baseline level of HIV knowledge, the positive change in HIV knowledge among participants represents an important foundation for behaviour change. The association between level of attendance and increase in HIV knowledge change score may support that attendance at the intervention was a predictor of changing HIV knowledge. These findings are in agreement with the body of evidence that Stepping Stones is effective in increasing HIV and STI knowledge (Skevington et al. 2013). Correct knowledge and perceived risk of acquiring HIV is an important component of reducing sexual risk practices, even if knowledge alone is insufficient (Bernardi 2002; Sayles et al. 2006; Tenkorang and Maticka-Tyndale 2014).

Gender was associated with HIV knowledge change scores, with young men having a greater score increase than young women when adjusting for covariates. Given that both young women and men had the same median score at baseline and that attendance was controlled for in the model, this variance may be due to observed gender differences in facilitation of the intervention. The peer facilitators were an important component of the intervention, and variance in their skills may have had an influence on the different HIV knowledge outcomes, which will be further examined in a future paper.

Our findings did reveal a small but non-significant increase in participants’ CUSE from pre- to post-intervention. We did not find a difference in reported CUSE between young women and men pre- or post-intervention. We found marginal changes in reported condom use among young men, albeit non-significant. In South Africa, young women participating in Stepping Stones and Creating Futures demonstrated a trend towards increasing condom use (Jewkes et al. 2014). Young women in the present intervention showed no change in the frequency of condom use and had a slight decrease in use at last vaginal sex from pre- to post-intervention. This may be due to the fact that condom use and sexual decision-making are typically under the control of men (Closson et al. 2018) and young women’s sexual partners may not be the young men participating in the intervention.

This study piloted an adapted intervention and did not include a control group, the sample size was small, the power to detect significant changes was limited, and therefore, the results should be interpreted with caution. Our results may also be prone to social desirability bias, as participants may have answered favourably or suppressed information on questionnaires. However, given the trusted relationship peer facilitators established with program participants, it is likely they felt comfortable answering honestly. Lastly, this study took place in one geographic location in Kenya where the research team has a long-standing relationship with the study population and therefore limits its generalizability to other settings.

Despite limitations, this study has strengths. This intervention occurred within an adolescent-friendly clinic and was implemented and facilitated by young people who grew up with street experiences. It reflects the ability of the intervention to function within the existing infrastructure, suggesting the potential for scale-up, sustainability, and validity in ‘real world’ circumstances.

Overall, this study has demonstrated that *Stepping Stones ya Mshefa na Kujijenga Kimaisha* program may be effective at increasing SCY’s HIV and condom-use knowledge, as well as health-seeking behaviours.

## Electronic supplementary material

Below is the link to the electronic supplementary material.

Supplementary material 1 (DOCX 21 kb)
