# Supplementary material for: Piloting an evidence-based intervention for HIV prevention among street youth in Eldoret, Kenya
Source: Int J Public Health. 2020 Apr 9;65(4):433–43. doi: 10.1007/s00038-020-01349-8 (PMC7275002; doi:10.1007/s00038-020-01349-8)
Supplement: Supplementary file 1 — Supplementary material 1 (DOCX 21 kb) [file 38_2020_1349_MOESM1_ESM.docx]

**Electronic Supplementary Material**

**International Journal of Public Health**

**Piloting an evidence-based intervention for HIV prevention among street youth in Eldoret, Kenya**

**Supplementary Table 1.** Attendance at pilot intervention stratified by gender and age groups in Eldoret, Kenya from 2017-18

|  | **N=80 Total**  **n (%)** | **Young women**  **N = 40** | | | **Young men**  **N=40** | | |
| --- | --- | --- | --- | --- | --- | --- | --- |
|  |  | **Total** | **Aged**  **16-19 Years**  **(n=20)** | **Aged**  **20-24**  **Years**  **(n=20)** | **Total** | **Aged**  **16-19**  **Years**  **(n=20)** | **Aged**  **20-24**  **Years**  **(n=20)** |
| **Attendance** |  |  |  |  |  |  |  |
| Median # of Sessions (IQR)  Range (0-24) | 11  (1-20) | 10  (1-18) | 9  (1-20) | 11  (3-16) | 15  (1-23) | 16  (3-23) | 7  (0-19) |
| **Attendance Level** |  |  |  |  |  |  |  |
| Low (0-7 sessions) | 33 (41.3) | 15 (37.5) | 9 (45.0) | 6 (30.0) | 18 (45.0) | 7 (35.0) | 11 (55.0) |
| Medium (8-16 sessions) | 20 (25.0) | 14 (35.0) | 4 (20.0) | 10 (50.0) | 6 (15.0) | 3 (15.0) | 3 (15.0) |
| High (17-24 sessions) | 27 (33.8) | 11 (27.5) | 7 (35.0) | 4 (20.0) | 16 (40.0) | 10 (50.0) | 6 (30.0) |

**Supplementary Table 2.** Sexual and health seeking practices pre- and post-intervention stratified by gender in Eldoret, Kenya from 2017-18

| **Sexual Practices** | **Pre-Intervention** | | | | | | **Post-Intervention** | | | |
| --- | --- | --- | --- | --- | --- | --- | --- | --- | --- | --- |
|  | **Total** N=80  n (%) | **Young women**  N = 40  n (%) | | **Young men** N=40  n (%) | **Baseline Retained^a^** N=67  n (%) | | **Total**  N=67  n (%) | **Young women**  N=33  n (%) | | **Young men**  N=34  n (%) |
| **Participants who reported ever having vaginal sex** | | | | | | | | | | |
|  | N=76 | N=39 | N=37 | | N=64 | N=66 | | N=33 | N=33 | |
| **Last Vaginal Sex Condom Use** | | | | | | | | | | |
| Yes | 27 (35.5) | 16 (40.0) | | 11 (27.5) | 23 (34.3) | | 23 (34.8) | 9 (27.3) | | 14 (42.4) |
| No | 49 (64.5) | 23 (57.5) | | 26 (65.0) | 41 (61.2) | | 43 (65.2) | 24 (72.7) | | 19 (57.6) |
| **How often do you use condoms for vaginal sex?** | | | | | | | | | | |
| Always / Most of the time | 14 (18.4) | 9 (23.1) | | 5 (13.5) | 12 (18.8) | | 15 (22.7) | 8 (24.2) | | 7 (21.2) |
| Sometimes | 41 (53.9) | 17 (43.6) | | 24 (64.9) | 33 (51.6) | | 36 (54.5) | 14 (42.4) | | 22 (66.7) |
| Never | 20 (26.3) | 13 (33.3) | | 7 (18.9) | 18 (28.1) | | 15 (22.7) | 11 (33.3) | | 4 (12.1) |
| Missing | 1 (1.3) | - | | 1 (2.7) | 1 (1.6) | | - | - | | - |
| **How many different people have you had vaginal sex with in the last month?** | | | | | | | | | | |
| 0 | 16 (21.1) | 0 (0) | | 16 (43.2) | 15 (23.4) | | 16 (24.2) | 1 (3.0) | | 15 (45.5) |
| 1 | 39 (51.3) | 27 (69.2) | | 12 (32.4) | 32 (50.0) | | 31 (47.0) | 20 (60.6) | | 11 (33.3) |
| > 2 | 21 (27.6) | 12 (30.8) | | 9 (24.3) | 17 (26.6) | | 19 (28.8) | 12 (36.4) | | 7 (21.2) |
| **Have you ever exchanged sex for money, shelter?** | | | | | | | | | | |
| Yes | 25 (32.9) | 21 (53.8) | | 4 (10.8) | 21 (31.3) | | 20 (30.3) | 19 (57.6) | | 1 (3.0) |
| No | 49 (64.5) | 17 (43.6) | | 32 (86.5) | 41 (61.2) | | 44 (66.7) | 13 (39.4) | | 31 (94.0) |
| Refuse Answer | 1 (1.3) | 1 (2.6) | | 0 (0) | 1 (1.5) | | 2 (3.0) | 1 (3.0) | | 1 (3.0) |
| Missing | 1 (1.3) | - | | 1 (2.7) | 1 (1.6) | | - | - | | - |
| **In the past month have you exchanged sex?** ^(Of those that have ever exchanged sex)^ | | | | | | | | | | |
|  | N=25 | N=21 | | N=4 | N=21 | | N=20 | N=19 | | N=1 |
| Yes | 16 (64.0) | 15 (71.4) | | 1 (25.0) | 13 (61.9) | | 18 (90.0) | 18 (94.7) | | 0 (0) |
| No | 9 (36.0) | 6 (28.6) | | 3 (75.0) | 8 (38.1) | | 2 (10.0) | 1 (5.3) | | 1 (100) |
| **How often do you typically exchange sex?** ^(Of those that have ever exchanged sex)^ | | | | | | | | | | |
| Daily | 9 (36.0) | 9 (42.9) | | 0 (0) | 8 (38.1) | | 13 (65.0) | 13 (68.4) | | 0 (0) |
| Weekly | 2 (8.0) | 2 (9.5) | | 0 (0) | 2 (9.5) | | 5 (25.0) | 5 (26.3) | | 0 (0) |
| Once a month | 4 (16.0) | 3 (14.3) | | 1 (25.0) | 2 (9.5) | | 0 (0) | 0 (0) | | 0 (0) |
| In the past, but not in the past 6 months | 7 (28.0) | 4 (19.0) | | 3 (75.0) | 6 (28.6) | | 1 (5.0) | 0 (0) | | 1 (100) |
| Don’t Know | 3 (10.7) | 3 (14.3) | | 0 (0) | 3 (14.3) | | 0 (0) | 0 (0) | | 0 (0) |
| Refuse answer | 0 (0) | 0 (0) | | 0 (0) | 0 (0) | | 1 (5.0) | 1 (5.3) | | 0 (0) |
| **Health Seeking Practices** | | | | | | | | | | |
| **Have you ever been tested for HIV? (N=80)** | | | | | | | | | | |
| Yes | 75 (93.8) | 37 (92.5) | | 38 (95.0) | 64 (94.0) | | 65 (97.0) | 32 (97.0) | | 33 (97.0) |
| Never | 5 (6.3) | 3 (7.5) | | 2 (5.0) | 4 (6.0) | | 2 (3.0) | 1 (3.0) | | 1 (3.0) |
| **When was the last time you were tested?** ^(Of those ever tested, n=75)^ | | | | | | | | | | |
|  | N=75 | N=37 | | N=38 | N=64 | | N=65 | N=32 | | N=33 |
| < 1 month | 31 (41.3) | 21 (56.8) | | 10 (26.3) | 25 (39.1) | | 25 (38.5) | 17 (53.1) | | 8 (24.2) |
| 1 to 3 months | 18 (24.0) | 2 (5.4) | | 16 (42.1) | 14 (21.9) | | 23 (35.4) | 6 (18.8) | | 17 (51.5) |
| 3 to 6 months | 8 (10.7) | 5 (13.5) | | 3 (7.9) | 7 (10.9) | | 8 (12.3) | 3 (9.4) | | 5 (15.2) |
| > 6 months / Don’t Know | 18 (24.0) | 9 (24.3) | | 9 (23.7) | 17 (26.6) | | 8 (12.3) | 6 (18.8) | | 4 (12.1) |

^a^ Participants with both baseline and endline data representing complete cases
